# Management of Hepatic Encephalopathy

**DOI:** 10.4061/2011/841407

**Published:** 2011-09-21

**Authors:** G. Wright, A. Chattree, R. Jalan

**Affiliations:** ^1^University College London Institute of Hepatology, The Royal Free Hospital, Pond Street, London NW3 2PF, UK; ^2^Department of Gastroenterology, King Georges Hospital, Barley Lane, Goodmayes, Ilford, Essex IG3 8YB, UK

## Abstract

Hepatic encephalopathy (HE), the neuropsychiatric presentation of liver disease, is associated with high morbidity and mortality. Reduction of plasma ammonia remains the central therapeutic strategy, but there is a need for newer novel therapies. We discuss current evidence supporting the use of interventions for both the general management of chronic HE and that necessary for more acute and advanced disease.

## 1. Introduction

There are a plethora of therapeutic approaches to targeting varying severities of hepatic encephalopathy (HE), the neuropsychiatric presentations of liver disease. There is a need for newer therapies for patients with advanced HE and worsening acute liver injury. Reduction of plasma ammonia remains the central strategy although novel strategies may be beneficial. We discuss current evidence supporting the use of therapeutic interventions for both the general management of chronic HE and that necessary for more acute and advanced disease.

## 2. General Management of Chronic Encephalopathy (Table 1)

### 2.1. Ammonia-Lowering Strategies

#### 2.1.1. Dietary Protein Supplementation

Patients with cirrhosis often have a poor nutritional reserve due to anorexia, poor diet, malabsorption, and altered metabolic state. Hospitalized patients are often hypermetabolic and hypercatabolic, worsened by complications such as gastrointestinal bleeding, continued anorexia, and fasting for tests. Yet dietary protein has the potential to drive further ammoniagenesis, and so previously dietary protein restriction was common practice. However, protein restriction is no longer advocated as does not improve HE and may be harmful [1]. In fact high-protein diets are well tolerated in cirrhotic patients [2], with consensus supporting the need for normal or high dietary protein (1–1.5 g/kg protein and 25–40 kcal/kg per day) [2, 3]. Rare exceptions arise occasionally with inborn errors of metabolism or acute liver failure (ALF) patients intubated for grade 3-4 HE associated with high circulating ammonia when protein restriction with maintained calorie intake (e.g., dextrose infusion) is necessary. 

#### 2.1.2. Branched-Chain Amino Acids (BCAAs)

BCAAs are chiefly derived from dairy products and vegetables and account for 25% of total dietary protein. They are a good substrate for protein synthesis, both conserving and restoring muscle mass in advanced liver disease. In cirrhosis, poor dietary intake leads to a deficiency of BCAA and resultant accumulation of aromatic amino acids, both worsening protein-energy deficits and glutaminergic neurotransmission (increased false neurotransmitter precursors). In “high protein diet” intolerant and severely malnourished patients, BCAA supplements may be useful to provide the necessary nitrogen intake without a decline in mental state, with vegetable proteins likely to be better tolerated due to their higher BCAA content. As BCAAs are under the influence of circulating insulin, the insulin resistance state of cirrhosis may limit there nutritional benefit unless systemic insulin replacement is implemented. However, a number of meta-analyses have failed to find consensus on the use of BCAAs in cirrhosis from a wealth of conflicting data [4, 5]. 

In most cirrhotic patients, a modified eating pattern, based on several meals and a late evening snack, is adequate [4, 6].

#### 2.1.3. Glycaemic Control

Disturbed glycaemic and lipid control is common in progressive liver disease and only worsened by the stress response in critically unwell patients. Therefore, once feeding has commenced, tight glycaemic control using insulin may be necessary to reduce oxidative stress (which triggers insulin resistance), limit mitochondrial liver damage, and improve endothelial activation (e.g., NO production), which will improve blood flow, limiting tissue injury, and improve outcome [7, 8].

#### 2.1.4. Vitamins and Nutrients

Cirrhosis also leads to deficiencies of lipid-soluble vitamins, minerals, and micronutrients. For example, Zinc is a cofactor in the urea cycle [9] and also found in vesicles of predominately glutamatergic presynaptic terminals thereby having a role in neurotransmission [10]. Zinc supplementation (600 mg/day) has been studied without obvious benefit though replacement should be considered if the patient is deficient [11]. Autopsy specimens from patients with hepatic coma and pallidal MR images of patients with HE suggest that manganese deposition in the basal ganglia may be a factor [12, 13]. However, as with earlier studies evaluating the role of gut bacterial products like mercaptans, phenols and medium- and short-chain fatty acids [14], there has been little cumulative evidence to support targeted treatment strategies.

## 3. Probiotics

Most of the ammonia produced by the gut is from the deamination of dietary amino acids by bacteria, with a small contribution from the urea produced by urease-positive bacteria. In the critically ill and malnourished patient, levels of the predominant defensive bacteria strains (Bifidobacterium and Lactobacillus) decline. Antibiotics may further lead to ammonia-producing bacteria ameliorating hyperammonaemia. Probiotics are living nonpathogenic microorganisms utilized as food ingredients that may have a role in the treatment of HE. Probiotics are thought to exert an effect in HE by reducing intestinal ammonia production by enterocyte glutaminase and reduce bacterial translocation, modulate proinflammatory responses, and modulate gut permeability [15]. Furthermore, probiotics bypass the small bowel and get fermented by colonic bacteria to form lactic, acetic, and butyric acids, and gas (mainly hydrogen); any resultant intestinal hurry may increase the expulsion of ammoniagenic bacteria. In randomized placebo controlled trials [16], probiotics have been shown to reduce gut ammonia production and inflammation [16, 17]. It is worth noting that fermentable fibres alone were also beneficial in that study. This is not unexpected as the common effect of probiotics, aside from a decline of substrate for other bacteria [18] and reduced translocation, is the fermentation of nonabsorbed sugars (e.g., mono-, di- and oligosaccharides). This fermentation of sugars leads to the production of differential amounts of lactic acid, ethanol, and CO_2_ to modulate intestinal acidity and gas production.

## 4. Purgatives

A purgative is an agent which cleanses the bowel by increasing the evacuation of luminal contents. This is beneficial in HE as it allows for reduced intestinal ammonia production and despite limited evidence from randomized controlled trials remain the most widely used therapy for HE.

### 4.1. Nonabsorbable Disaccharides

It is unclear how non-absorbable disaccharides exert a beneficial effect. There have been many proposed mechanisms (1) enhanced growth of nonurease-producing bacteria [19], (2) catharsis secondary to bowel acidification reducing ammonia absorption [20, 21], (3) proliferation of healthy bacteria by providing additional carbohydrate and thus nitrogen (even as ammonia) into protein, and/or (4) providing carbon and energy and so spare bacterial ammonia metabolism [22]. More specifically, lactulose (a sugar) passes through the small bowel completely undigested (unlike glucose, sucrose, and lactose, which are easily fermented in the small bowel). Once in the colon, lactulose is fermented by anaerobic bacteria, especially *Bacteroides spp*. Fermentation of lactulose by colonic bacteria yields important weak acids (lactic, acetic & butyric) and gases (e.g., hydrogen). This leads to the acidification of ammonia into ammonium which is poorly absorbed. However, physiologically a total daily dose of 10–20 g is small compared to 500–1000 g faeces/day, such that the impact on acidity/reduced faecal pH on the faecal flora is likely to be limited. This is supported by the failure of mannitol and sorbitol, which both cause low pH, to improve HE [23]. The production of colonic hydrogen may be more important as only 7 g of lactulose produces 1 Litre of hydrogen that could induce intestinal hurry and shift massive amounts of colonic bacteria [24]. However, it may be the provision of energy in preference to ammonia that accounts for the benefit of non-absorbable disaccharides.

A comprehensive meta-analysis of non-absorbable disaccharides has suggested that current data from randomised clinical trials do not support its routine use in clinical practice [25] though newer clinical studies suggest benefit with lactulose conferring improved neuropsychometric and quality of life scores [26], which lends weight to the overwhelming amount of anecdotal evidence that disaccharides are beneficial. It is likely that the impact of other therapies initiated at the same time often confounds any benefit on HE severity by the established ammonia-lowering effect of non-absorbable disaccharides. 

Compliance, adverse effects, clinical safely, and cost effectiveness are necessary concerns. It is often overlooked that aggressive use of lactulose causes significant gaseous distension, discomfort, and diarrhoea which may lead to poor compliance. Furthermore, frank dehydration, prerenal uraemia, hyponatraemia, or aspiration of lactulose can occur. Therefore, although non-absorbable disaccharides are relatively cheap, their cost effectiveness should be balanced against clinical outcomes.

### 4.2. Other Purgatives

Enemas are beneficial as a means of expelling ammonia-producing gut flora by both cleansing and colonic acidification [27] but are no better than oral purgatives like lactulose. Therefore, if bowel motions can be maintained at ≥2/day, then enemas may not offer any additional benefit.

## 5. Nonabsorbable Antibiotics

The contribution of intestinal urease-positive bacteria to gut ammonia production is mainly in the colon rather than gastric mucosa (e.g., Helicobacter pylori), due to their number and more alkaline colonic pH which favours enhanced ammonia diffusion, such that Helicobacter pylori eradication has no therapeutic benefit [28]. Oral, non-absorbable, synthetic antibacterial agents such as Neomycin and Rifaximin have been used to inhibit the growth or kill susceptible ammoniagenic bacterial species, showing comparable efficacy to lactulose [29]. Rifaximin is a synthetic antibiotic related to rifamycin, with wide antibacterial activity against both aerobic and anaerobic gram-negative and gram-positive bacteria. In random controlled studies Rifaximin is proven efficacious (maintaining remission and reducing hospitalization with HE even in patients already on lactulose), and a superior safety profile and thus preferred to neomycin [30, 31], Although beneficial, non-absorbable antibiotics are often reserved for patients who fail to respond to non-absorbable disaccharides.

## 6. Modulators of Interorgan Ammonia Metabolism (Figure 1)

The concept of manipulating endogenous biosynthetic pathways to eliminate nonurea waste nitrogen as a substitute for defective urea synthesis is well established [32]. Despite abnormal urea-cycle functioning, reducing total body nitrogen by promoting the synthesis of non-urea nitrogen-containing metabolites with high excretion rates appears to be of benefit.

### 6.1. Arginine Supplementation

L-arginine is an important dietary substrate for the urea cycle which allows for ammonia detoxification to urea (via arginase). L-arginine is a semiessential amino acid, as although metabolically produced, in some disease states may require dietary supplementation. In cases of the childhood urea cycle disorders (e.g., deficiency of argininosuccinate synthetase (AS) and argininosuccinase (AL)), dietary restriction of L-arginine triggers the rapid development (15–68 hours) of symptomatic hyperammonaemia (e.g., vomiting, lethargy, or irritability) [33]. In these disorders, there is a significant reduction in urea production, with nitrogen instead accumulating as mainly glutamine, ammonium, and to a limited extent alanine and glutamate. In AS and AL deficiency, the provision of additional dietary L-arginine promotes the synthesis of citrulline and argininosuccinate, allowing for the urinary excretion of nitrogen. 

In ALF, systemic hypotension and cerebral oedema may be associated with increased plasma nitric oxide (NO) levels. L-arginine is the rate-limiting substrate for NO production but is deficient in ALF due possibly to increased arginase activity in the liver which converts it to urea and ornithine. There have been no clinical studies evaluating a role for L-arginine supplementation in HE, though animal studies suggest that correcting L-arginine deficiency may alter portal hypertension and cerebral oedema via arginase-dependent reduction in hyperammonaemia and/or NO-dependent mechanism(s).

### 6.2. Phenylbutyrate

Phenylbutyrate (converted to phenylacetate in vivo) is an established therapy for hyperammonaemia associated with urea cycle disorders [34], which are characterized by elevated glutamine levels. This excess can be mopped up by phenylacetate, which covalently combines with circulating glutamine to form renally excreted phenylacetylglutamine, removing glutamine as a substrate for ammoniagenesis. So far phenylbutyrate has proved ineffective in the treatment of HE associated with liver failure, probably because a high glutamate state, a prerequisite for phenylacetate to work, is absent in liver failure. 

### 6.3. Sodium Benzoate

Similarly sodium benzoate increases the renal excretion of ammonia but as hippuric acid (hippurate), the glycine conjugate of benzoic acid [32]. Sodium benzoate also improves the encephalopathy with inborn errors of metabolism [35] and is as effective as lactulose in the treatment of acute portosystemic HE [36]. 

### 6.4. Combined Intravenous Sodium Phenylbutyrate and Benzoate (Ammonul, Ucyclyd Pharma)

In urea-cycle disorders, combination therapy results in a 79% reduction in plasma ammonia, and 84–98% improved survival with late onset disease, though poor in neonates and high peak ammonia values [37]. If untreated, only 16% of neonates survive, compared to 72% with late onset disease [38]. However, as the N-acyltransferases that conjugate glutamine to phenylacetate and glycine to benzoate are located in the liver and kidney, the severe hepatotoxicity of ALF may eventually lead to response failure, especially with the saturation of enzyme capacity (e.g., phenylacetate to PAG) [39, 40].

### 6.5. L-Ornithine L-Aspartate (LOLA)

LOLA provides L-ornithine and L-aspartate as substrates for glutamate production in muscle leading to a reduction in circulating ammonia and in models of liver failure further suggest that LOLA reduces brain oedema of advanced HE [41]. In a double-blind randomized control study of cirrhotics with mild HE, one week of LOLA reduced ammonia and improved mental function [42]. A cross-over study showed that 20–40 g/day of infused LOLA ameliorated postprandial increases in ammonia following oral protein loading [43]. However, at higher doses, this study increased plasma glutamate, unchanged glutamine, and increased urea production [43] contradicting the muscle ammonia detoxification hypothesis. Furthermore, 40g dosing induced hyperglycaemia and hyperinsulinaemia [43]. As yet, there are no studies in patients with ALF, and its use in ALF is currently not recommended. Critically, there are concerns that the ammonia-lowering effects of LOLA may only be transient, due to rebound hyperammonaemia on stopping LOLA [44], as a significant rise in glutamine levels eventually becomes a source for ammoniagenesis by the kidney and intestines (through glutaminase) [45]. Additionally, aspartate is unlikely to offer added benefit as in animal models it failed to reduce ammonia [46].

### 6.6. L-Ornithine Phenylacetate (OP)

OP is a novel therapy targeting interorgan ammonia and amino acid metabolism [44]. OP reduces toxic levels of ammonia by ornithine acting as a substrate for glutamine synthesis from ammonia in skeletal muscle. This combination unlike other therapies targeting interorgan ammonia metabolism (e.g., LOLA), by stopping the recycling of ammonia (trapped as ornithine-glutamine) via phenylacetate excreting the ornithine-related glutamine as phenylacetylglutamine in the kidneys. It has been shown to correct the hyperammonemic state in animal models of cirrhosis [47] and ALF [48], limiting brain oedema and rises in ICP. Clinical studies are currently underway.

## 7. Others

### 7.1. Acarbose

The hypoglycaemic agent acarbose which stimulates gut motility, through the inhibition of intestinal glucose absorption by promoting intestinal saccharolytic bacterial flora in preference to proteolytic flora, thereby reducing substrate for ammonia production. In a cross-over randomized trial of cirrhotic patients with low-grade HE and type-2 diabetes mellitus, 8 weeks of acarbose (100 mgs TDS) significantly decreased ammonia blood levels, and intellectual function, aside from decreasing fasting and postprandial glucose, and reducing glycosylated haemoglobin levels [49]. However, acarbose is unlikely to be an option except in those with coexistent type-2 diabetes mellitus. 

### 7.2. Bromocriptine

Bromocriptine, a dopamine agonist, has been used with limited success for disturbances in dopaminergic neurotransmission associated with chronic intractable HE [50, 51], but such studies failed to show a clear benefit over standard therapy [49]. Furthermore, in cirrhotic patients with ascites, it can induce hyponatraemia [52]. However, there is anecdotal evidence to suggest a benefit in a small number of cirrhotic patients with low-grade encephalopathy and basal ganglia injury with associated dopamine deficiency.

## 8. Correction of Precipitating Factors (Table 2)

Worsening encephalopathy is often precipitated by a number of factors which can be anticipated and promptly corrected. Though HE may be triggered by uncommon events, it is important to outline the management of the more common precipitants.

### 8.1. Constipation

Enemas are beneficial as a mean of expelling ammonia producing gut flora either due by cleansing or colonic acidification [27]. However, there is only limited evidence to show a benefit over the use of oral purgatives like lactulose. Therefore, if bowel motions can be maintained at ≥2/day, enemas are only used as an adjunct to the primarily used non-absorbable disaccharides. 

### 8.2. Infections

bacterial infections predispose to variceal bleeding in cirrhotic patients. A meta-analysis of antibiotic use in variceal bleeding reported a 30% decrease in rate of infection and 9% improvement in short-term survival [53, 54]. Septic encephalopathy may also confound or mimic HE.

### 8.3. Gastrointestinal Bleeding

Due to the high-protein content of blood and thus nitrogenous load, there is increased intestinal ammonia production. This ammoniagenic blood meal and precipitation of HE are potentially related to an absence of the branched-chain amino acid isoleucine which protects the inhibitory effect of ammonia on the TCA cycle in neuronal cells. 

### 8.4. Portosystemic Shunts

Persistent shunts may account for worsening HE poorly responsive to standard oral therapies and may be best treated by shunt closure. 

### 8.5. TIPSS Insertion

The creation of a portosystemic shunt (used to stabilize patients with uncontrolled variceal bleeding or intractable ascites) may induce HE (especially within the first few months). Prophylaxis against encephalopathy with Lactitol (60 g/day) or Rifaximin (1200 mg/day) is not proven to be effective during the first month after TIPSS [55]. Therefore, careful selection of patients for a TIPSS or surgical shunt is necessary.

## 9. Acute Severe HE: Intracranial Hypertension and Cerebral Oedema (Table 1)

ALF is characterised by rapid onset HE with cerebral oedema and intracranial hypertension and progression to coma stages, independently associated with a 30% mortality [56]. Early ventilation, intensive care unit admission and judicious use of available therapies have led to a significant decline in deaths as a result of cerebral oedema. Aiding liver recovery by prompt and specific treatment of the cause of acute liver injury, treating precipitating factors such as dehydration, electrolyte and acid-base imbalance, [57], infection [58], and ameliorating hyperammonaemia remain at the forefront of therapy. The following therapeutic strategies are utilized in the management of severe HE requiring ventilation.

## 10. General

Early airway maintenance is necessary to protect the airway and prevent high carbon dioxide tension and hypoxia which can result in cerebral hyperaemia [59]. Sedation and mechanical ventilation is also essential to safely manage agitation. Once intubated, the head should be elevated by 10–20° with minimal intervention and care when moving patients and optimize intracranial pressure (ICP) without compromising the cerebral perfusion pressure [60, 61]. Airway protection will also reduce the likelihood of aspiration, pneumonia, defective gas exchange, and infection. Sedative requirements (e.g., fentanyl, midazolam, or propofol) are low with worsening severity of HE but are likely to increase with recovery. Propofol is a useful sedative because it will reduce ICP, and because of its nonhepatic metabolism will not accumulate. It may, however, induce hypotension [62].

### 10.1. Circulatory Support and Fluid Management

ALF is a hyperdynamic state with high cardiac output, low mean arterial pressure, and low systemic vascular resistance [63]. Generalized vasodilatation, which produces profound activation of the neurohormonal system, culminates in vasoconstriction of regional vascular beds [64]. Mean arterial pressure should be maintained at a level to keep the cerebral perfusion pressure between 50 and 65 mmHg [65]. The onset of multiorgan failure often necessitates the use of inotropes. Circulatory failure often becomes refractory to inotropes and up to 70% of patients die [66]. A routine short synacthen test on admission to guide the use of steroids is important as adrenal insufficiency is a common complication of ALF [67]. 

### 10.2. Renal Support

Renal dysfunction is common due to either prerenal, hepatorenal, or nephrotoxic (e.g., acetaminophen) injury [68]. This frequently requires renal replacement [66] with continuous (compared to intermittent) haemofiltration [69]. This avoids rapid water shifts seen with intermittent therapy [70], providing greater haemodynamic stability and improved cerebral perfusion pressure [69, 71]. Furthermore, due to impaired hepatic lactate metabolism, lactate-free dialysates are preferred [72].

### 10.3. Electrolyte Imbalance

Electrolyte imbalance should be corrected aggressively. Hyponatraemia ≤125 mmol/L may precipitate cerebral oedema and is a contraindication for orthotopic liver transplant (OLT) [73, 74]. Induced hypernatraemia has been shown to improve ICP and reduce inotropic requirements in traumatic brain injury and ALF [75].

### 10.4. Antimicrobial Agents

The incidence of sepsis in ALF is a significant factor in mortality and a contraindication to transplantation. Around 75% develop bacterial and 30% fungal infections [76, 77]. The administration of broad-spectrum antibiotics/antifungal therapy should be initiated at the first sign of infection, with focused treatment once the organism is identified. Despite the absence of randomized control trials of prophylactic systemic antimicrobials in ALF, their use is widespread [78, 79].

### 10.5. Glycaemic Control

Both hyper- and hypoglycaemia need rapid correction as they may worsen brain oedema. The role of tight glycaemic control in ALF has not been ascertained but must be instituted with caution because of the tendency for the development of hypoglycaemia.

## 11. Specific

### 11.1. Mannitol

Mannitol (an osmotic diuretic) increases brain capillary osmolality, drawing water from the brain tissue into the capillaries, and has been shown to significantly reduce the extent of cerebral oedema and improve survival [80, 81]. Bolus doses of 20% mannitol at 1 g/kg are preferred. Plasma osmolality should be kept <320 Osm/L, as mannitol is less effective with increasing osmolality. If patient is oliguric, mannitol may accumulate and can only be used with concomitant haemofiltration.

### 11.2. Dexamethasone

In ALF, reducing inflammation (whether systemic or local) by utilizing the anti-inflammatory effects of steroids may improve cerebral haemodynamics and prevent/treat intracranial hypertension [79, 82, 83]. However, trials using dexamethasone in advanced ALF have shown little effect on the frequency of cerebral oedema or survival [80].

### 11.3. Mild Hypothermia

In models of ALF, induced hypothermia significantly reduces brain water, duration of encephalopathy, and improved outcome [84–86]. Using cooling blankets to induce moderate hypothermia (target core temp. 32–33°C) can lead to a reduction in ICP, even in patients unresponsive to mannitol and/or ultrafiltration [87, 88]. Hypothermia also significantly improves cardiovascular haemodynamics with reduced noradrenaline requirements [88], likely related to a reduction in arterial ammonia and also brain ammonia extraction and flux [87, 89]. As yet, there is no data from randomized control trials on the use of hypothermia in ALF but is worth considering in patients with uncontrolled intracranial hypertension.

### 11.4. Thiopental Sodium

By inducing cerebral vasoconstriction through the inhibition of nitric oxide synthetase, intermittent bolus injections of thiopental (1.5–3.5 mg/kg) reduce elevations of ICP [90]. However, its use is limited to intractable increases in ICP unresponsive to other therapies. Because of profound negative effects on systemic haemodynamics, its use is limited.

### 11.5. Indomethacin

Nonsteroidal anti-inflammatory (NSAIDS) may modulate brain function [91] (with possible effects on cognitive function via modulation of the glutamate-nitric oxide-cyclic GMP pathway [92]). Indomethacin (0.5 mg/kg), a nonselective cyclooxygenase inhibitor [93], can reduce ICP and cerebral oedema independent of a change in cerebral blood flow [94]. However, its use is limited by nephrotoxicity, platelet dysfunction, and risk of gastrointestinal bleeding. Poor brain penetration of NSAIDs at therapeutic levels requires high doses which increases the risk of toxicity [92, 95].

### 11.6. Antiepileptic Drugs (AED's)

In some ALF patients with grade 3-4 HE, subclinical seizures occur, and the use of phenytoin was shown to significantly reduced seizure frequency and the development of increased ICP [96]. 

### 11.7. N-Acetylcysteine (NAC)

In a case of acetaminophen overdose, NAC must be continued irrespective of the time between the overdose and presentation and acetaminophen level as it can prevent the progression of ALF and reduces mortality especially in those who progress to grade III–IV HE [97]. There is less convincing evidence for NAC in nonacetaminophen overdose [98, 99]. In nonacetaminophen ALF, NAC may improve survival by its effects on cardiac output, oxygen extraction and consumption, and due to its antioxidant effects that ameliorate the significant oxidative stresses that occur with liver failure.

### 11.8. Flumazenil

In a large placebo controlled trial focusing on intensive care patients with advanced HE (grade III-IV), the short-acting benzodiazepine-receptor antagonist flumazenil was shown to rapidly improve the neurological score in 15% and electroencephalogram (EEG) findings in 30% of patients within minutes of its administration [100]. However, flumazenil does not lead to any lasting effect or correct HE, unless coadministered with a long-acting therapy [101], and as such is not recommended.

## 12. Liver Support and Transplantation

### 12.1. Transplantation

Transplantation offers definitive intervention for liver failure with a swift return to a normal mental state though minimal HE may persist in a few due to some as yet unknown irreversible cerebral changes [102]. Disparity between donor organs and recipients has led to a plethora of extracorporeal liver assist devices [103, 104] and even partial hepatectomy [83, 105] to aid or supplant the failing liver. 

### 12.2. Extracorporeal Liver Assist Devices

Such devices may be either “*biological*” (using either immortalised cultured hepatocytes or whole animal livers), or “*nonbiological*” (using extracorporeal blood purification to dialyse albumin-bound hydrophobic substances), ultimately mimicking endogenous excretory and synthetic liver function. The extracorporeal devices under clinical evaluation include the following.

*Molecular Adsorbent Recirculating System (MARS). *It provides counter-current haemodialysis against albumin and bicarbonate circuits.

*Single-Pass Albumin Dialysis (SPAD)*. It provides counter-current albumin dialysis against high-flow blood in a fibre haemodiafilter, which unlike MARS is discarded after passing the filter. As it uses a standard renal dialysis device, continuous venovenous haemodiafiltration is possible.

*Prometheus system.* It provides direct albumin adsorption with high-flux haemodialysis after selective filtration of the albumin fraction through a specific polysulfone filter. 

All devices successfully remove protein-bound toxins but have variable effects on systemic (versus portal) haemodynamics, and the potential to worsen coagulopathy. The clinical benefit of such devices is unclear but may at least offer a bridge to either transplantation or liver recovery. 

## 13. Conclusion

Ammonia-lowering therapy remains at the cornerstone of standard medical care for HE, along with measures to treat precipitating factors and specific interventions for the cerebral sequelae of advanced disease. Understanding interorgan ammonia metabolism and the pathophysiological basis of HE are most likely to lead to the development of new therapeutic approaches [45]. However, there is a lack of conclusive evidence from clinical studies even for current best practice [25, 106] and, therefore, a requirement for robust randomized controlled trials to drive a more evidence-based approach.

## Figures and Tables

**Figure 1:**
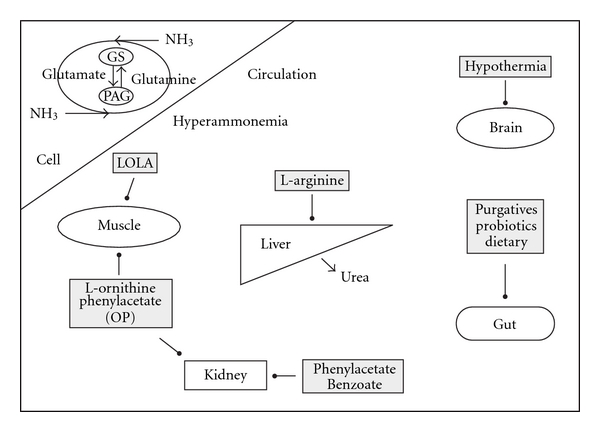
Therapies manipulating interorgan ammonia and amino acid metabolism. In liver failure, the relative activities of cellular glutamine syntheses (GS) and phosphate-activated glutaminase (PAG) in different organs influence interorgan ammonia and amino acid metabolism. With a loss of hepatic urea cycle capacity, hyperammonaemia is predominately due to worsening intestinal and renal ammonia efflux, with skeletal muscle having the potential to increase its ability to detoxify ammonia. Though the brain also detoxifies ammonia, this is counterproductive as resultant astrocyte glutamine accumulation induces brain swelling. This schematic highlights not only current standard therapies for hyperammonaemia which principally act on individual organs (e.g., purgatives targeting intestinal ammonia production), but also newer interventions targeting multiple organs (e.g., LOLA and OP).

**Table 1:** Treatment stratagems used in HE.

HE grade: I-II
General management

Hyperammonemia
Dietary protein supplementation
Purgatives
(i) Nonabsorbable disaccharides
(ii) Enemas
Non-absorbable antibiotics
Modulation of interorgan ammonia
(i) L-ornithine, L-aspartate (LOLA)
(ii) Sodium benzoate
(iii) Phenylacetate
Others
(i) Flumazenil “Bromocriptine” acarbose
Emerging therapies
(i) Probiotics

HE grade: III-IV

Cerebral edema & elevated ICP

General
(i) Ventilate
(ii) Sedate (e.g., Propofol)
Specific
(i) Antimicrobials
(ii) Hypertonic saline
(iii) Mannitol
(iv) Dexamethasone
(v) Induced hypothermia
(vi) Thiopentone
(vii) Indomethacin
(viii) Antiepileptic drugs (AED*'*S)
(ix) N-acetylcysteine (NAC)

Transplantation

Orthotopic liver transplant (OLT)
Partial hepatectomy
Liver assist devices

**Table 2:** Precipitating factors in hepatic encephalopathy.

Precipitating factors in HE
Constipation
Dehydration
Gastrointestinal bleeding
Infection
Excessive dietary protein
Hypokalaemia
Hypoglycaemia
Hypothyroidism
Hypoxia
Metabolic alkalosis
Anaemia
Azotaemia/uraemia
Medications (narcotics, sedatives, etc.)
Hepatoma
TIPS, surgical shunt
Vascular occlusion
